# Correspondence

**DOI:** 10.12669/pjms.35.3.1071

**Published:** 2019

**Authors:** Mukhtiar Baig

**Affiliations:** *Prof of Clinical Biochemistry, Medical Educationist, Faculty of Medicine, Rabigh, King Abdulaziz University, Jeddah, Saudi Arabia*

**Usage and types of mobile medical applications amongst medical students of Pakistan and its association with their academic performance**

I read the above titled manuscript by Dr. Aliya Hisam et al published in in March-April 2019 issue of Pakistan Journal of Medica Sciences. It’s a very interesting study and I was particularly more interested because it was very similar to the study published by me. 1

I have two queries regarding this research. Firstly, the authors mentioned that “Independent sample t-test was applied to assess association between average usage of mobile medical application and academic performance and a p-value of <0.05 was taken as statistically significant.” Literature indicates, “the independent t-test, is an inferential statistical test that determines whether there is a statistically significant difference between the means in two unrelated groups.” 2 So I want to ask how did the authors calculate the association by applying Independent sample t-test? For assessing association, we need different types of statistical tests.^3^

Bjornsen et al., (2015) and Lepp et al., (2014) have calculated the association of mobile phone use and students grades by using the correlation and regression analysis.^4^,^5^

Secondly, authors have mentioned in the conclusion that “Association between average usage of mobile medical applications and academic performance of the students was statistically significant.” However, in the discussion section, they did not discuss this important finding. Surprisingly, there was not a single sentence mentioned about this. Being a reader, I was curious to know the authors’ viewpoint regarding this association.

***Response from the authors:*** Thank you for taking interest in the research article. Following is the response to your queries:

**First query:**

The students were divided into two groups based on mobile application usage: one group using mobile application routinely and other group not using mobile application routinely. Then the groups mean academic performance in the most recent examination was compared among the two groups.

About 323 (72%) students whose were routinely using medical application scored 69±7% in their professional examination while 125 (28%) students, who were not using medical application scored 67±9%. The association between average usage of medical application and academic performance was statistically significant (p<0.01).

I could have applied correlation or regression in case I had both variable as continuous that is hours or minutes utilized in using medical mobile application and academic performance (marks). But instead I had two groups i.e. routinely using mobile applications and not routinely using mobile application so t test was applied.

**Second Query:**

In discussion section, it would have been good if we had discussed the association of medical mobile application with academic performance but to best of our knowledge we could not find a study to which we could relate our study results and the available ones full text was not approachable. Also because of word count limitation by the Journal we had to skip this point.


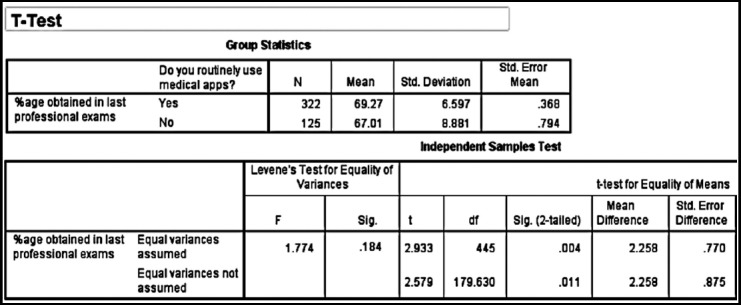


Dr. Aliya Hisam

Associate Professor, Department of Community Medicine, Army Medical College (AMC), Faculty Member National University of Medical Sciences (NUMS), Rawalpindi, Pakistan

Thank you for sharing the response of the authors to my queries related to their published manuscript. However, I am sorry to say their response is not scientifically sound.

***Query 1:*** For their response to one of the query regarding the use of independent sample t-test to assess association between average usage of mobile medical application and academic performance. The authors have not provided any reference for improving the knowledge of the readers that describes that independent sample t-test can be applied to assess association between two variables.

The results authors have shown in their response letter are also showing the mean difference, not the association. Actually, in the manuscript, authors found the mean difference by applying independent t-test and they labeled that association, which is not correct.

***Query 2:***As the authors mentioned that “to best of our knowledge we could not find a study to which we could relate our study results and the available ones full text was not approachable,” but the unavailability of related literature is not a hurdle in discussing their finding and probable reasons behind their finding. Furthermore, it would have been better if they had mentioned this point in the discussion that related literature is not available so they cannot correlate their study results with others.

Authors have also mentioned the word count limitation by the journal for skipping this point. It was one of the main study outcome that was necessary to discuss because they mentioned this outcome in the title and results. Moreover, 3000-3500 words are sufficient to describe any study results.

Prof. Mukhtiar Baig

The discussion on this topic is now closed- Chief Editor
